# Physician management of immunoglobulin in Canada: A qualitative study

**DOI:** 10.1371/journal.pone.0354817

**Published:** 2026-08-19

**Authors:** Kelly Holloway, Umair Majid, Quinn Grundy

**Affiliations:** 1 Institute of Health Policy, Management and Evaluation, University of Toronto, Toronto, ON, Canada; 2 Canadian Blood Services, Toronto, ON, Canada; 3 Lawrence Bloomberg Faculty of Nursing, University of Toronto, Toronto, ON, Canada; Tekirdag Namik Kemal University: Tekirdag Namik Kemal Universitesi, TÜRKIYE

## Abstract

**Background:**

The uses of plasma-derived medical products like Immunoglobulin (Ig) have expanded in recent years. This increasing demand has prompted countries globally to undertake efforts to both increase plasma sufficiency, and develop mitigation measures for shortages in supply, including engaging in demand management.

**Methods:**

We conducted among the first qualitative studies of physician management of Ig in Canada. We used semi-structured interviews and analyzed data with interpretive thematic analysis.

**Results:**

Our findings reflect perspectives from 12 clinicians who prescribe Ig in Canada in the context of rising use of and demand for this treatment. Across the sample, we identified three general orientations toward Ig prescribing: 1, concern and uncertainty about prescribing Ig; 2, good stewardship of a scarce resource; 3, advocacy for access to treatment.

**Conclusion:**

This study examined how physicians who prescribe Ig understand their practice in relation to the rising international demand for, and cost of this treatment. To date, policy efforts focusing on physician prescribing have encouraged a rational and evidence-based approach to prescribing. Our study complicates that approach. We identified three general orientations toward Ig prescribing. These should not replace rational prescribing but suggest that a mechanistic approach solely focused on the rational application of the best evidence could be inadequate.

## Introduction

The uses of plasma-derived medical products (PDMP) like Immunoglobulin (Ig) have expanded in recent years [1]. This increasing demand reflects advancements in medical science, the broadening scope of treatable conditions, and demographic shifts [2]. It has prompted countries globally to undertake efforts to both increase plasma sufficiency [3,4], and develop mitigation measures for shortages in supply [5], including engaging in demand management [6,7]. Given the central role physicians play in prescribing and managing these therapies, their perspectives on and experiences treating patients with Ig require greater attention. Physicians who care for people with rare diseases for which Ig may be effective also contend with the challenges around Ig procurement and administration that arise from the broader context of global challenges related to the supply of plasma and the cost of PDMP production. Understanding physicians’ experiences and insights is crucial in shaping cost-effective, equitable and sustainable treatment strategies, particularly as Ig therapy emerges as both a critical and costly resource in healthcare. As the demand for Ig increases, there are government calls for ‘rational prescribing’ of Ig [1,8]. While a rational and evidence-based approach to prescribing is important, our attention to the social components of how Ig is managed can complicate and contextualize the challenges of implementing this approach.

In Canada, immunoglobulins are plasma protein products regulated by Health Canada as a drug. The country’s national blood operators, Canadian Blood Services (CBS) and Héma-Québec (H-Q) collect recovered plasma from whole blood donations, and source plasma from volunteer plasma donors plasma for Ig. CBS and H-Q send this plasma to fractionators for production of plasma protein products, which are returned to the blood operators for use in Canadian patients. The plasma collected in Canada is inadequate to meet the needs of Canadian patients. Therefore, plasma protein products are also purchased to meet the demand. These products are largely produced from paid plasma donors. Canada’s blood supply chain is a single, integrated system operated on behalf of the provincial and territorial governments [9]. CBS manages a national formulary of plasma protein and related products on behalf of provincial and territorial ministries of health [9]. Ig products listed in the CBS or H-Q formularies are funded through provincial and territorial tax dollars as part of the Canadian universal healthcare system, and the provinces and territories monitor Ig utilization [10]. Regional guidelines have been developed to respond to an increase in demand for Ig [11], but there are variations among these guidelines, and there is no national standardized protocol [12]. There are efforts underway to address how prescribing in Canada can be more evidence-based, streamlined and cost-effective [10,11].

Existing approaches to Ig management do not sufficiently address the ways in which the practice of prescribing involves interpersonal relationships between physicians and patients and political, economic and cultural contexts [13]. In exploring these factors, we trouble dominant ideas about how Ig should be managed. Sociologists have pointed to the fact that 'rational prescribing' can be an idealised and technical framework that does not always account for the socially situated practice of prescribing [13]. Clinical practice is not always determined by the best evidence but also by social dynamics such as patient demands, physician habits, and pharmaceutical marketing [14]. There is very little scholarship addressing how physicians navigate complex decisions about prescribing this therapy. This qualitative study aims to understand how physicians prescribing Ig in Canada manage Ig treatment amid public discussions about cost, sufficiency and potential shortages.

## Methods

In this qualitative study, we conducted in-depth, semi-structured interviews with physicians who prescribe Ig in Canada. This study received ethics approval from the University of Toronto Research Ethics Board (44593) and the Canadian Blood Services Research Ethics Board (2023.021). All participants provided verbal consent before participating in the study and they were not compensated. Verbal consent for participation and audio-recording was sought instead of written consent to reduce administrative burden given that interviews were arranged and conducted online. The study is reported in accordance with the Consolidated Criteria for Reporting Qualitative Research (COREQ) checklist [15].

### Participant selection and recruitment

We used a purposive sampling strategy, a qualitative sampling technique used to select participants with experience of the phenomenon to provide an in-depth understanding. We recruited physicians in Canada who had treated adult patients with Ig in Canada through organizations representing relevant specialties in consultation with CBS Stakeholder Engagement. See Table 1 for a list of organizations that received the recruitment email invitation; prospective participants contacted the PI to arrange interview.

**Table 1:** List of organizations that received the recruitment email.

Organizations that represent relevant disease areas	National Advisory Committee on Blood and Blood Products Immunodeficiency Canada Canadian Society of Allergy and Clinical Immunology Clinical Immunologists Network – Canada Canadian Hematology Society Canadian Neurological Sciences Federation Canadian Rheumatology Association Canadian Dermatology Association Canadian Society of Transplantation

### Data collection

Interviews took place on Zoom from April to September 2024. They were conducted by KH, the lead researcher of the study, who has a PhD in sociology and is a Scientist with CBS, and lasted 30–80 minutes. The semi-structured interview guide was iteratively refined across the interviews.

**Fig 1:**
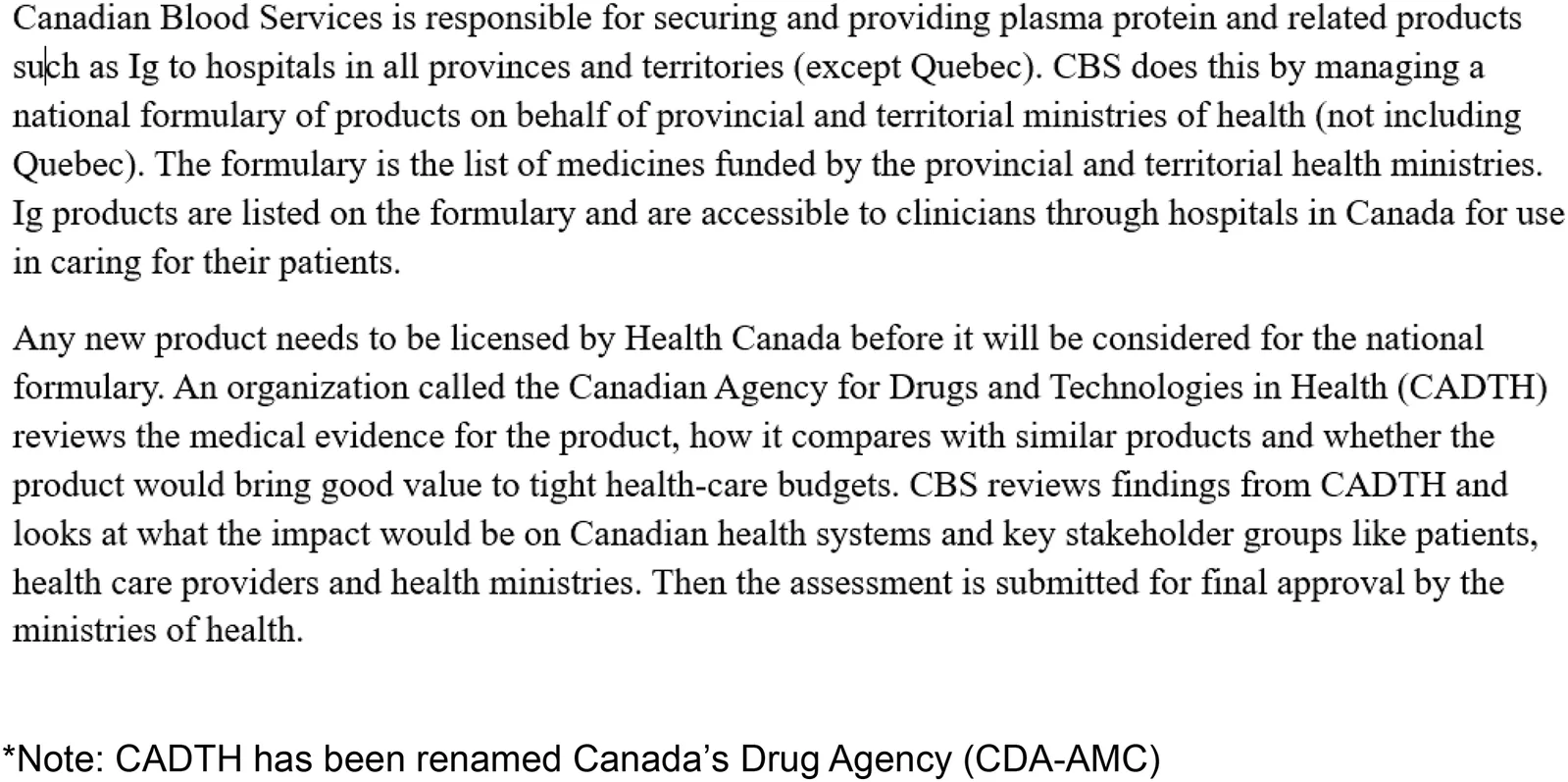
Short Description about is distributed in CanadaIg’s health system. *Note: CADTH has been renamed Canada’s Drug Agency (CDA-AMC).

Participants were asked questions about their practice, relationships with other specialists, the illnesses they treat, their knowledge of Ig and what they communicate to patients about this treatment, and their process for prescribing Ig. Participants were also asked policy questions around availability of Ig, guidelines, and the blood service’s formulary development process for making decisions about which products are covered by health insurance in Canada. Participants were given a short explanation about how Ig is distributed in Canada’s health system (Fig 1: Short description) and asked to reflect on this system.

### Data analysis

KH, the lead researcher, and UM, a Research Assistant on the study conducted an iterative interpretive thematic analysis [16] involving a careful reading of transcripts, coding in NVivo14 (a qualitative data software), and continual reflection about the data in relation to literature in the field and our own perspectives and roles within this system. We generated themes on different aspects of clinician experiences with prescribing Ig as a treatment, which were then discussed and developed with a third member of the study team, QG. Our analysis focused on understanding the meaning conveyed by participants about topics explored in the interviews. UM, the research assistant, conducted initial line-by-line coding and wrote research memos summarizing each participants’ account organized by their answers to the interview, such as conditions they treat, knowledge of Ig, etc. The lead researcher and the research assistant then reviewed the codes and started to organize them into themes, while also adding contextual information from the memos offering more depth about each participants’ account. Broad thematic categories were developed from this process after conducting eight interviews (research on Ig, patient awareness of Ig, care of patients with Ig, cost of Ig, and access to Ig). To further understand the extent to which these themes remained meaningful with more participants, we then conducted four more interviews. Following this, we reviewed the final transcripts in relation to the themes until we gained a sufficiently comprehensive understanding of the issues raised in the data to offer meaningful qualitative insight about this nascent area of study, a process to achieve what is called meaning saturation [17]. These broad themes were then discussed and refined by the research team and put into dialogue with the literature informing the study to generate our analysis. Given our focus on the participants’ decisions about prescribing, we use the concept of ‘orientation’ sociologically to mean the ways in which beliefs, values and experiences continually inform how physicians approach their clinical practice. Our analysis seeks to situate the prescribing physician a person who both draws on their fundamental beliefs and values, and responds to the existing context, to navigate the complex field of prescribing this particular therapy. All 12 participants are represented in the analysis. Each orientation was derived from patterns of reasoning that recurred across the full sample and contributed to the construction of these orientations. Quotations were selected to illustrate each analytic point in participants’ own words.

This analysis process also involved reflection about the fact that the lead researcher is an employee of CBS, and recruitment was facilitated by CBS. Thus participants could have been more reserved about offering critical feedback about this organization and also particularly sensitive to defending their role as good stewards of the blood supply. Given that this population is not easy to reach, the interviewer’s role also could have been the factor that facilitated trust and access to the participants. The other members of the research team provided an outside perspective to help contextualize this data and think about how this is a particular sample of the broader population of prescribing physicians, a point we raise in the limitations section. Authors resolved disagreement about analysis through dialogue. We offer thick description and the use of verbatim quotations to establish the credibility of our analysis [18].

## Results

We interviewed 12 physicians that prescribe Ig in Canada (See Table 2 for demographic information). Because specialists who prescribe Ig in Canada are a small inter-connected group of people, to protect participants’ confidentiality, we do not offer specifics about their treatment area or demographic information, except to indicate the range of specialties, practice and location (see Table 1).

**Table 2:** Characteristics of the sample (n = 12).

Characteristic	N
Specialty Immunology Hematology Neurology Rheumatology	4 4 3 1
Years practicing 10 to 20 years <10 years >20 years	6 3 3
Geographic location Ontario British Columbia Alberta	7 3 2

Our findings reflect perspectives from clinicians who prescribe Ig in Canada in the context of rising use of and demand for this treatment. Across the sample, we identified three general orientations toward Ig prescribing; these are not profiles of individual prescribers but composites, reflecting the range of experiences with Ig management. The three orientations toward Ig management reflect three primary foci, expressed through participants’ understandings and practices, and constructed from the collective analysis of interviews: 1, concern and uncertainty about prescribing Ig; 2, good stewardship of a scarce resource; 3, advocacy for access to treatment.

### Concern and uncertainty about prescribing Ig

Despite treating patients with Ig, participants expressed continued uncertainty about why Ig worked better in some patients over others with the same disease, why some patients responded better to subcutaneous immunoglobulin (SCIg) or to intravenous immunoglobulin(IVIg), why certain patients had side effects while others did not, or how long they should treat a patient with Ig. Participants also expressed uncertainty about optimal dosage and frequency. Thus, they acknowledged uncertainty and explained that managing this uncertainty required continual interaction with the patient, paying careful attention to how the patient responded to treatment.

This uncertainty did not come from a lack of training, but rather from a lack of research on Ig as a treatment – on the mechanisms, side-effects, and other indications that could require Ig. Further, given the complexities around diagnosing rare conditions, which do not always have a straightforward clinical diagnosis pathway, specialist participants had experience treating patients who were first misdiagnosed or had gone long periods without diagnosis. One participant explained,

“Immunology as a whole; you might get one or two courses in medical school. You can; you could go very, very deep into immunology and still not understand it. […] The immune system is complicated for many people. And I wouldn’t expect most physicians to truly understand the complexities of it. Including family physicians” (P1).

Participants described situations where the patient received a diagnosis in their community, were referred to another specialist for their condition, and received ineffective—and in hindsight, inappropriate—treatment. These difficulties in diagnosis could lead to a delay in treatment, prolonging patients’ symptoms, and were exacerbated in rural locations and areas underserved by specialists with experiences treating these rare conditions. Further, some patients received Ig, but did not understand that it could be for a limited duration. Participants explained that once a patient was on Ig, it was difficult to suggest that the treatment was no longer necessary. A participant explained that they struggled with telling patients they should no longer be on Ig:

“I do find it a bit frustrating for many reasons, most, the primary one which is resource allocation but also, the message that a patient gets when they’re on a treatment that they don’t need, is very hard to un-tell. So, it puts you in a very awkward position” (P8).

Acknowledging that many family physicians had not even heard of some of these rare conditions for which Ig was indicated, one participant said they provided education to family physicians on red flags that would require a referral to their specialty. Participants also tried to communicate with physicians in their network about appropriate use of Ig.

### Good stewardship of a scarce resource

Participants understood Ig as a scarce and expensive resource. They learned about the expense of this treatment in their training, and all said they made decisions about prescribing carefully, considering the cost of this treatment. Some discussed cost with patients to engage them in cost-effective care. Others refrained, believing that the medication was necessary for their patient, and that a discussion of cost could cause undue stress. Others exercised caution through discussions with their patients about dose and duration of treatment – depending on the underlying condition, some explicitly communicated to patients that Ig is a bridging rather than ongoing therapy. One participant said if they have a patient that has been on a high dose of Ig for ten years, they will suggest moving to incrementally lower doses. Participants also thought about cost of Ig as a treatment in relation to alternative medicines that are as effective and not as expensive. One participant explained, they do not hesitate to prescribe Ig if it is appropriate. At the same time, “if there are less expensive options that don’t necessarily have more side effects, then I, you know, I try to keep that in mind” (P4).

The orientation toward Ig management as a form of good stewardship arose most obviously when the interviewer introduced information about how Ig is distributed in Canada (See Fig 1) and asked participants if they were familiar with this process, and whether they thought that it served the needs of patients. Given our recruitment strategy, many participants were familiar with the process, and some had even been involved in developing policies to address potential shortage of Ig in Canada, as well as other policy issues related to management of this treatment such as payment for plasma. Some identified as not only a physician but as a resource utilization steward for blood products. Or as one participant put it, they needed to be “reasonable gatekeepers of this public resource” (P2).

They acknowledged that good stewardship of Ig required more communication and training among physicians. Some participants said they tried to communicate with other specialists in their area about not using too much Ig, and using it in the right clinical context. For example, one participant suggested that community neurologists should be calling specialists who are more connected to the academic community to seek guidance on the appropriate us of Ig:

“I’d like to communicate that, we’re using too much of it, and, we should be using it in the right clinical context. So, if you’re not sure about the medication, just call us, and we can have a conversation about it” (P5).

In some provinces, SCIg has been introduced as an effort to mitigate issues of cost and improve access. Some participants saw this modality as advantageous for patients because hospital-based infusions are inconvenient and it was potentially risky for a person who is immunocompromised to enter a hospital for infusion. Some also thought it was less costly for health systems. One participant started to recommend SCIg because they noticed that IVIg was having an impact on infusion capacities in their hospital: “A lot of the patients have to come in, like, on a monthly basis. And, so, that’s a huge impact on the infusion capacities of the hospital" (P11). Many were trying to determine how the introduction of SCIg fit into the broader landscape of the health system, and also figure out whether and how it should be integrated into the care they provide.

### Advocacy for access to treatment

Participants in this study were very much aware of how their practice was a part of a broader health system, which is dynamic and political. They reflected on the impact of the cost of increasing use of Ig on healthcare budgets, access to specialists for people in rural and remote areas, and training of primary care physicians. Further, they often acted as advocates for their patients to get access to Ig by engaging with various levels of hospital administration and government. They were engaged at a policy level with respect to management of Ig as a blood product, being good stewards, and participating in policy planning about issues like potential shortage of Ig. One participant explained:

“As a haematologist, I’m advocating for resources for my patient, and then as a transfusion doc, of course I’m advocating for the patient, but then I’m also advocating for the system and trying to balance those things, and I think that that’s where things like IVIg come in. I think you have more contact with the Ministry who’s concerned about, you know, the cost of IVIg over time” (P11).

This kind of advocacy required participants to be responsible for not only patients but also resource utilization.

Participants indicated that they thought carefully about how their role was intertwined with political matters. Our description of the process for distributing Ig indicated that part of this process includes deliberation about whether any new product would bring “good value to tight health-care budgets” (see Fig 1). Some participants cautioned the use of that phrase, pointing out that “good value” had different meanings depending on where one is situated in the healthcare system (as a regulator, a government agency, a physician, a patient, or a pharmaceutical company). Some expressed concern about the role of pharmaceutical companies in these processes who lobby to have their product covered on the formulary. For example, one participant had been privy to a situation where a company was arguing that their product should be covered because it would compete with IVIg, but they had not done a head-to-head trial. Further, participants suggested the phrasing around “good value” inherently assumed scarcity: “I think when you use the word ‘tight healthcare budgets,’ it already suggests that we’re coming from a place where we’re in scarcity” (P5). The participant explained, “So, it should maybe be something like, ‘good value to our sustainable long-term healthcare budget,’ or something like that. You know, using words that are more neutral, because this word is not neutral” (P5).

Several participants had been part of discussions about potential shortages of Ig, because there was a need to make policy decisions about how to prioritize medications in that scenario. One participant explained that physicians learn about procurement, availability, and manufacturing because of efforts to plan for potential shortage:

“We’re part of the discussion when those threats loom, I think appropriately, and it’s part of, like, trying to gauge who, who really needs it. Who could wait an extra month. Uh, thankfully, I guess we’ve just been very lucky that it hasn’t materialized where we’d have to start to triage in that way” (P6).

Even outside of the context of a potential shortage, not every IVIg product was approved in the national formulary, and sometimes a product was phased out of the market. This could be disruptive for patients, A participant explained:

“I think inside the blood banks and the transfusion medicine, they have to manage that much more where,[…] they’re changing products, [patients are] not getting the same product over all the time, and, those patients who are, have reactions that some products, or have trouble, trouble tolerating some" (P7).

Physicians acted as the gatekeeper to treatment, and while they were not always at the table for decisions about coverage, they had to manage care for patients when a treatment became unavailable, or when treatments changed because of a formulary decision. They therefore became invested in decisions about coverage and allocation of resources.

Part of playing an advocacy role for their patient involved navigating a complex health system with multiple players. For instance, the introduction of SCIg came with new players and systems that participants had to understand and manage. Participants articulated that the availability of SCIg removed the need for patients to come to hospital for infusion, but some expressed concern about how that shift would affect dosing, benefits, harms, and effectiveness, questioning whether the shift was driven by clinical need, health system priorities, or commercial interest. A participant articulated their uncertainty about what is driving this change:“I don’t know where the money is going. I don’t know where the profit’s going. I don’t know where the push is” (P3). They described a patient with an immune deficiency who could get a lower dose of Ig through SCIg at home, and not be exposed to potential infections at the hospital. While this is advantageous in some ways, the participant worried about the potential risk of administering high-dose Ig injections at home and the unknowns around the impacts, benefits, or harms of the subcutaneous formulation.

Participants recognized the need for support in the community to help patients transition to SCIg and administer injections at home: “You kind of need a program behind it, and, I haven’t quite figured out who that’s gonna be for all of the patients that we have here” (P6). In response to this need, participants explained that the pharmaceutical companies that produce SCIg had created and funded “patient support programs,” which included injection training, while simultaneously encouraging physicians to use their brand of SCIg. Some participants were cautious about those relationships with pharmaceutical companies, indicating that they preferred to pay attention to the evidence and expressing concerned about the effectiveness of these programs. One participant said working with these programs was challenging because they required a high degree of coordination, including detailed direction about Ig administration from the prescribing physician. This could be challenging; for instance, this participant said neurologists often did not have all of the relevant information about the patient, as it was predominantly a nursing scope of practice, and wished instead to work with specialist nurses in the publicly-funded system. Another participant echoed this concern indicating that many of their patients had not transitioned to SCIg because there were too many difficulties transferring to a manufacturer-led patient support program. This participant emphasized that because patient support programs are brand exclusive, patients had to move from a nurse they trusted to administer IVIg in the hospital, to a program that required them to stop working with that nurse and potentially switch products.

“Because the patient support programs are product-specific, the patients weren’t able to just keep working with the same nurse, they had to switch programs entirely, and so that was super-disruptive to them. They kind of had a relationship with one person, and then, were no longer able to work with that person” (P2).

Participants described the burden of the transition to SCIg not just on patients, but also on their practice’s office, given the coordination involved with the company, or multiple companies’ patient support programs. Some suggested it would be most effective to have a nurse who worked with the immunodeficiency program in the hospital to help coordinate transition to SCIg because they had the condition-relevant expertise, injection skills and training and could provide the social support that was necessary to manage treatment: “the problem with SCIg is if, is it really requires more, potentially more of an infrastructure […], to deliver it, because it’s being delivered at home, that means it requires patient training, and education” (P7). For example, the transition required careful consideration of whether the patient had someone who could work with them to do the injection or the hand dexterity to do it on their own.

## Discussion

Given increasing demand for Ig worldwide and the concurrent challenges with collecting sufficient plasma to meet demand, it is important to understand the perspectives of the physicians who practice at the intersection of the systems that collect and distribute this treatment, and the patients who receive it. Smith’s (2025) review and analysis of sociology of prescribing draws our attention to the symbolic power of prescribing as “a liminal space for enacting and ordering health” [13]. Our study demonstrates that the work that takes place during that clinical interaction between physician and patient for Ig is a critical point of knowledge exchange, negotiation of power, and enactment or disruption of policies and processes organized through healthcare systems and blood services. While there is some literature pointing to how physicians manage Ig [2], there is very little in-depth investigation into their experiences prescribing this in-demand biologic treatment. This qualitative study offers insight into physicians’ understandings of Ig, some of the social and political factors they consider when prescribing this treatment, and the context in which they practice. It also offers an analysis of how they develop orientations toward Ig prescribing to make sense of this context, which can be leveraged to make decisions about prescribing. This involves a negotiation between responding to the needs of their patients and managing a scarce and costly resource.

Our investigation advances scholarship on Ig management and sufficiency and on integration of biologics for rare disease into health systems. Our study also offers insight into why efforts such as evidence-based guidelines, rationalized prescribing and increased governance could be challenging to implement. For instance, while participants in this study who were specialists in their area felt they had adequate training and knowledge about Ig, and about the cost of this treatment, they were not sure that other physicians, particularly family physicians had sufficient knowledge or experience with the conditions they treat, or Ig as a treatment. This finding resonates with work indicating a potential gap in education and awareness on immunodeficiencies, neuropathies and Ig therapies among primary care physicians in the US [19,20]. We are hesitant to suggest that this be one more topic to add to training of family physicians. Rather, as participants in this study suggest, specialists could play a role in educating their colleagues in their specialty and beyond, to ensure appropriate use. This educational effort was not necessarily focused on adherence to guidelines, but about basic knowledge of the illnesses treated, and when and how to refer to the right specialist. Further study could explore how this outreach is undertaken by physicians who are doing this educational work, what kind of time and resources are required to support this work, and whether family physicians see this as helpful.

Resonant with some of the work on the sociology of prescribing, our study demonstrates that prescribing immunoglobulin is a socially situated practice, shaped through structural, institutional and interpersonal dynamics [13]. These findings complicate the reliance on rationalized prescribing as a policy solution, which has been problematized in scholarship in this area. For instance, a study of chronic care management in Denmark found that attempts to rationalize prescribing by informing GPs about drug effectiveness, safety and price did not satisfy their knowledge needs. GPs wanted to “understand how medications co-operate with patients’ bodies and everyday life” [14] (p 115). Wadmann and colleagues argue that the knowledge needs of GPs encompass “practice-relevant knowledge,” “a form of knowledge that enables clinicians to handle the daily clinical work” [15]; (p 115). Practice-relevant knowledge is not counter to scientific knowledge, but rather, encompasses more than evidence. In our study, participants pointed to the knowledge they gained through engaging with their patient and modifying the treatment carefully in response to the patients’ reactions. They especially leaned on this clinical knowledge where they found the evidence lacking. Importantly, they also pointed to the need for more research – on questions that came out of their clinical practice. They found that patients responded differently to different dosages, and that side-effects varied significantly. These factors are not well studied, which is especially relevant as physicians navigate the introduction of SCIg.

The sparse literature comparing the use of IVIg and SCIg [21,19] is sometimes driven by the companies that produce SCIg, and does not offer any context to the history of these modalities and what they represent for physicians. This study uniquely offers insight into physicians perspectives on the introduction of SCIg: the feeling that it has been pushed by the companies that create it or by health system leaders who think it will save money; the difficulties with transitioning care to pharma-funded and disease-specific patient-care programs; and the social supports that are necessary to manage Ig at home. These insights intersect with emerging work on patient support programs [20]. These programs, like the ones organized to support patients using SCIg, are becoming more common. Ten percent of the prescription drugs currently marketed in Canada are attached to such a program, concentrated around brand-name, branded generic, biologic and high cost drugs, often for rare diseases [20]. Given the points raised by participants in this study about these programs, we concur with recommendations for more transparency and independent evaluation of their role [20].

In short, prescribing Ig is a complex socially mediated task which requires being aware of the patient, the evidence, the health system and the policy landscape. Findings from our study resonate with emerging literature in the sociology of prescribing, pointing to the need for more research exploring this phenomenon. Smith’s (2025) reflections on the future domains of a sociology of prescribing (care, power, expertise, and work) are instructive. Applied to the topic of the management of Ig, questions of care include the power-relationship between prescriber and patient when it comes to diagnosis and prescription, how interprofessional team dynamics impact prescribing, and the various ways of enacting care through validating and treating patients to uphold expectations of care. Our earlier research indicates that recipients of Ig have difficulty navigating diagnosis and treatment of their rare disease [22]; this aspect of care at the intersection of patient and clinician is a critical area for future study, and should involve not just physicians but nurses, informal caregivers, and even non-human actors in this system [13] such as guidelines, diagnostic technologies, and the treatment itself. Particularly as this treatment is framed as a scarce and costly resource, scholarly attention to power dynamics in health systems is crucial.

Our earlier work [23] also indicates that recipients of Ig are aware of conversations about shortages and cost and are thus uncertain and concerned about their future access to this treatment. This is another area in which the interaction with the physician is a tense social negotiation of needs versus expertise which takes place in the midst of a broader discussion about domestic sufficiency of plasma. This case requires careful attention to how expertise is claimed and enacted. Scholarship on prescribing has documented how it is entangled with technical knowledge, guidelines, applied practice, as well as professionalism and pharmaceutical marketing practices [13,14]. While these dynamics surfaced in our study, each deserves more focused attention.

Finally, prescribing is work; professional work in health systems is dynamic and subject to labour practices, health financing, technologies, medical education, boundaries, specialization, and regulation [13]. Our findings regarding education about Ig, remote and rural access to specialists, healthcare budgets and interactions with the companies that produce and market Ig must be considered in the context of political and economic changes. Our study speaks to the boundaries of the work involved with delivering Ig intersects with the challenges involved in managing biologics for rare disease in existing health systems.

### Limitations

This study represents a small sample of experts from different fields of medicine, practicing in Canada. Their perspectives and experiences differed by disease area, which is not explored here because of the limited sample but represents an area for future research. Given that they were recruited with the help of our colleagues in CBS stakeholder engagement, and that the lead author and interviewer works for CBS, this sample is potentially unique. Participants were very aware of the policy landscape for managing Ig in a potential shortage, and some described themselves as good stewards of blood products in Canada. Despite efforts to reach physicians across the country, another limitation is that participants were from Ontario, British Columbia and Alberta, which only represents three of Canada’s thirteen provinces and territories, and specifically does not cover Quebec, which has a distinct healthcare jurisdiction and blood operator.

## Conclusion

This study examined how physicians who prescribe Ig understand their practice in relation to the rising costs of this treatment internationally. To date, policy efforts focusing on physician prescribing have encouraged a rational and evidence-based approach to prescribing. Our study complicates that concept by offering an in-depth qualitative analysis of physicians’ prescribing experiences in Canada, identifying 3 general orientations toward Ig prescribing: 1, concern and uncertainty about prescribing Ig; 2, good stewardship of a scarce resource; 3, advocacy for access to treatment. These orientations do not replace rational prescribing but suggest that a mechanistic approach solely focused on the rational application of the best evidence could be inadequate. Attention to this work and further research into this topic could strengthen efforts to address sustainability and domestic sufficiency for Ig into the future.

## Supporting information

S1File COREQ Checklist_Ig physicians. (DOCX)
