# Correction: Physician management of immunoglobulin in Canada: A qualitative study

**DOI:** 10.1371/journal.pone.0358848

**Published:** 2026-09-18

**Authors:** Kelly Holloway, Umair Majid, Quinn Grundy

S1 File was uploaded incorrectly. Please see the correct S1 File here.

## Supporting information

S1 File COREQ Checklist_Ig physicians. (DOCX)
